# Molecular profiling of the tumor microenvironment in glioblastoma patients: correlation of microglia/macrophage polarization state with metalloprotease expression profiles and survival

**DOI:** 10.1042/BSR20182361

**Published:** 2019-06-20

**Authors:** Marko Gjorgjevski, Ricarda Hannen, Barbara Carl, Yu Li, Emilie Landmann, Malte Buchholz, Jörg W. Bartsch, Christopher Nimsky

**Affiliations:** 1 Department of Neurosurgery, Philipps University Marburg, Baldingerstr., 35033 Marburg, Germany; 2 Clinic for Gastroenterology, ZTI, Philipps University Marburg, Hans-Meerwein Str. 3, 35043 Marburg, Germany

**Keywords:** Glioblastoma, M1/M2 polarization, macrophages, Metalloproteases, microglia, tumor microenvironments

## Abstract

Due to poor prognosis of glioblastoma (GBM), there is an urgent need to develop new therapeutic strategies. Besides eliminating GBM tumor cells and stem cells, a novel therapeutic approach aims to target Glioma-associated microglia/macrophages (GAMs). We investigated the molecular profile of GAMs correlated with patient prognosis by exploiting M1/M2-like polarization markers in a cohort of 20 GBM patients. Using quantitative PCR (qPCR), the markers CXCL10 (M1) and CCL13 (M2) were validated in human macrophages and applied to a global analysis of GBM tissue. Furthermore, proteinase genes, known to be associated with GBM progression (*ADAM8, MMP9, MMP14, ADAM10, ADAM17*), were analyzed in correlation to M1/M2 markers. Notably, expression levels of *ADAM10* and *ADAM17* are significantly correlated with an M1-like phenotype and are positively associated to patient survival. Whilst ADAM8 mRNA expression was equally correlated with M1- and M2-like markers, genes for *MMP9* and *MMP14* are significantly associated with an M2-like phenotype and association to impaired prognosis in the GBM patient cohort. Thus, we provide a robust and reliable combination of qPCR markers to characterize global microglia/macrophage status and the associated proteinase profiles in GBM patients that can be used to analyze the tumor microenvironment, the patients’ prognosis and preselect those GBM patients for which targeting the microglia/macrophage population by repolarization might be beneficial.

## Introduction

Microglia as resident immune cells of the central nervous system together with peripheral macrophages, recruited by tumor cells from circulating blood [7], constitute the most common cell types in the glioblastoma multiforme (GBM) microenvironment [4,12]. The fact that microglia/macrophages are significantly more abundant in GBM than in low grade gliomas (LGG) [4,12] may be indicative for their active role in glioma progression. Though the abundance of microglia/macrophages is of interest, it is their gradual polarization state that determines the pathophysiological character of this cell population. However, since macrophage polarization is considered to be a continuum between the M1- and M2-like phenotype [9,12], defining molecular markers for a reliable distinction between these two opposing phenotypes has proven to be challenging. For GBM, the concept of macrophage polarization is under debate, so that for instance in mouse models of GBM, a distinct molecular profile for Glioma-associated microglia/macrophages (GAMs) was described [18]. It remains to be determined whether a similar molecular profile applies for GAMs in human GBM.

Since M2-like microglia/macrophages are more resistant towards therapy in GBM, it is likely that adjuvant therapy even selects for M2 macrophage populations [11]. M2-like macrophages have been shown to increase growth of GBM cells *in vitro* by secretion of key signals such as Interleukin (IL)-6 [5] as well as tumor proliferation and motility by secretion of IL-10 [8], collectively leading to a failure to exert an anti-tumor activity and rather support tumor progress and invasion [20,22]. Thus, targeting M2-like macrophages, for example by a repolarization/reprogramming approach within GBM, could cause a significant improvement of prognosis and slowing of the disease [15]. It is therefore of clinical interest to identify the overall population of microglia/macrophages in GBM.

Apart from expression of M1/M2 markers in GAMs, zinc-dependent proteases of the metzincin superfamily such as MMPs (matrix metalloproteinases) or ADAM (A Disintegrin and Metalloprotease) proteinases are important modulators of the tumor microenvironment so that distinct populations of GBM-associated microglia/macrophages might be characterized by specific protease profiles in GBM [19,20]. Since some of these proteases (ADAM8, 10, and 17, MMP 2, 9, and 14) were shown to be associated with microglia/macrophage functions, profiling these protease genes in conjunction with M1/M2 polarization markers in GBM could provide novel insights into the molecular signature of these cells and might prove beneficial as diagnostic tool and predictor of patient survival.

## Materials and methods

### Human GBM samples

Ethical approval was obtained from the local ethics committee (file number 185/11), to collect tumor tissue samples from patients who underwent surgical resection of GBM after giving written informed consent. All selected patients underwent a gross total resection of a primary GBM WHO° IV, IDH wildtype and had adjuvant combined chemo- and radiotherapy. Before inclusion, we confirmed that the death cause of GBM patients stems from tumor progress, excluding other treatment complications or a secondary diagnosis that might provide false results on the microglia/macrophage population due to general inflammatory conditions. We included 17 male and 3 female patients with a mean age of 61.2 years (range 39−73 years), further data such as pathological characteristics are summarized in Table 1.

**Table 1:** Description of the patient cohort, including gender, age, and general condition quantified by Karnofsky and Eastern Cooperative Oncology Group (ECOG) Score at time of initial diagnosis, tumor location, and size

Sex	Age (years)	Karnofsky score	Initial ECOG score	Tumor location	Tumor size (mm × mm × mm)	MGMT promotor methylation status	p53 accumulation	KI67Li	EGFR vIII	Survival (days)
m	65	80	1	Right temporal	30 × 30 × 30	Methylated	Sporadicly accumulated	>10%	n.a.	189
f	51	70	1	Left trigonal postcentral	22 × 39 × 26	Not methylated	Moderately accumulated	5%	−	446
m	71	80	1	Right parietal	43 × 46 × 48	Methylated	Strongly accumulated	50%	+	533
m	69	80	1	Left parietal	30 × 30 × 30	Not methylated	Sporadicly accumulated	6%	−	297
m	67	90	0	Right frontal	40 × 40 × 40	Methylated	Strongly accumulated	30%	−	728
f	66	100	0	Right temporal	17 × 20 × 16	Methylated	Moderately accumulated	50%	−	1278
m	63	80	1	Right frontal	20 × 22 × 20	Not methylated	Focally accumulated	30%	+	724
m	53	90	0	Left occipitomesial	17 × 28 × 18	Not methylated	Strongly accumulated	10%	+	512
m	73	90	0	Right trigonal	47 × 47 × 38	Methylated	Weakly accumulated	20%	−	503
m	59	80	1	Right temporal	23 × 33 × 33	Methylated	Strongly accumulated	20%	−	562
m	61	70	1	Left parietal	40 × 40 × 40	Methylated	Focally accumulated	20%	−	361
m	67	80	1	Left frontolateral	20 × 30 × 30	Methylated	Moderately accumulated	30%	−	225
m	46	100	0	Left central	31 × 45 × 42	Methylated	Moderately accumulated	30%	+	857
m	57	90	0	Left temporal	39 × 35 × 17	Methylated	Weakly accumulated	10%	−	834
m	72	90	0	Left temporal	17 × 39 × 19	Methylated	Strongly accumulated	20%	+	661
m	66	90	0	Left temporal	37 × 50 × 44	Not methylated	Strongly accumulated	20%	−	649
m	45	80	1	Left temporal	31 × 28 × 35	Not methylated	Sporadicly accumulated	30%	−	577
m	72	90	0	Left temporal	30 × 40 × 43	Not methylated	Moderately accumulated	Up to 50%	−	387
m	40	90	0	Left temporal	28 × 34 × 31	Not methylated	Weakly accumulated	30%	−	544
f	70	90	0	Right postcentral	22 × 35 × 27	Methylated	Moderately accumulated	Up to 20%	−	328

All patients showed expression of wildtype isocitrate dehydrogenase (IDH), further parameters such as promotor methylation status of the O^6^-methylguanine-DNA-methyltransferase (MGMT), p53 accumulation, Ki67 Labeling index (Ki67Li), and expression of epidermal growth factor receptor (EGFR) variant III are listed above as well as survival time as days between initial diagnosis and death.

### Cultivation and polarization of human macrophages

THP1 human monocytic cells were cultivated in RPMI 1640 with 10% FCS. Cells were split after reaching a density of 1 million cells per ml. For polarization into M1-/M2-like phenotypes, 500000 THP1 cells were seeded in 2 ml RPMI containing 10% FCS per six-well. PMA (stock concentration 10 ng/μl) was added to each well to a final concentration of 10 ng/ml and cells were further cultured for 48 h. After 2 days, cells became adherent. The medium was aspirated, cells were washed with PBS and 2 ml fresh RPMI including 1% FCS was added. All polarization experiments were performed according to standard procedures: for polarization into M2-like phenotype, 20 ng/ml human IL-4 was added; for polarization into M1-like phenotype, 50 ng/ml LPS and 20 ng/ml human IFNγ were added. After 6 h of incubation the medium was changed to RPMI containing 1% FCS. After 3 days of cultivation, the cells were lysed for qPCR analysis. Peripheral blood monocytic cells (PBMCs) were isolated from buffy coats by density gradient centrifugation using Bicoll (Biochrom GmbH, Berlin, Germany). Briefly, 30 ml whole blood was layered on top of 10 ml Bicoll before samples were centrifuged at 1500 ***g*** for 30 min without breaks. The resulting band of mononuclear cells was isolated and washed twice with PBS. Cells were subjected to an elutriation with a JE-5B Rotor isolating only monocytes. For polarization, cells were first seeded at a density of 5 million cells per six-well in attachment media (Promocell, Heidelberg, Germany) for 2 h. Cells were washed and medium was changed to macrophage base medium (Promocell, Heidelberg, Germany) containing 2% AB serum (Merck KGaA, Darmstadt, Germany) for M0, 10 ng/ml GM-CSF (Peprotech GmbH, Hamburg, Germany) for M1 and 10 ng/ml M-CSF (Peprotech GmbH, Hamburg, Germany) for M2 phenotype. After 7 days of cultivation with 50 ng/ml IFNγ (Peprotech GmbH, Hamburg, Germany) for M1 and 20 ng/ml IL-4 (Peprotech GmbH, Hamburg, Germany) for M2 further activation factors were added. After three additional days in culture, cells were lysed and RNA was isolated.

### RNA isolation and qPCR

Frozen tumor tissue samples were thawed on ice, 50 mg tissue was homogenized in 1 ml QIAzol Lysis reagent (Qiagen GmbH, Hilden, Germany) using a disperser. After adding 200 μl of chloroform samples were vortexed vigorously and incubated 3 min at room temperature. Following a centrifugation of 15 min at 4°C the upper aqueous phase was transferred, 500 μl isopropanol were added and samples were incubated for 10 min at room temperature to precipitate RNA. After another centrifugation the pellet was washed with 75% ethanol and dried at 37°C before resuspending in water.

2 μg of RNA were transcribed into cDNA with the RNA to cDNA EcoDry Premix (Clontech, Saint-Germain-en-Laye, France) according to manufacturer’s instructions. qPCR was performed using a StepOne-Plus Real-Time PCR instrument (Applied Biosystems, Thermo Fisher Scientific, Dreieich) and Sybr Green in form of the Precision FAST MasterMix with ROX (Primer Design, Southhampton, U.K.). Primers for macrophage makers as well as proteases were validated QuantiTect Primer Assays (Qiagen GmbH, Hilden, Germany). PCR amplification reactions were carried out in 20 μl reaction volumes with 20 ng of cDNA. PCR conditions were: initial denaturation at 95°C for 10 min, followed by 40 amplification cycles at 95°C for 15 s and 60°C for 1 min. Samples were analyzed in triplicates and results were averaged. The mRNA for acidic ribosomal protein RPLP0 (primer name XS13) served as an internal reference gene for all real-time PCR reactions. Cycle time (Ct) was calculated by StepOne Software v2.0 (Applied Biosystems). For each gene, the 2^−ΔΔCt^ method [16] was performed to analyze relative quantities.

### Data analysis of the cancer genome atlas (TCGA)

Analysis of the TCGA dataset was performed using the browser of the University of Zurich (tcgabrowser.ethz.ch). For glioblastoma, a dataset of 159 RNAseq samples is available. The median expression level for analysis of overall survival was set to 50% for all genes analyzed. Only *P*-values of *P*<0.1 were considered to be significant.

## Results

### Validation of macrophage markers in polarized THP1 cells and blood derived PBMCs

To test the validity of qPCR markers for macrophage phenotype analysis, we used THP-1 cells and PBMCs as two independent macrophage-like cell types for our analyses.

**Figure 1:**
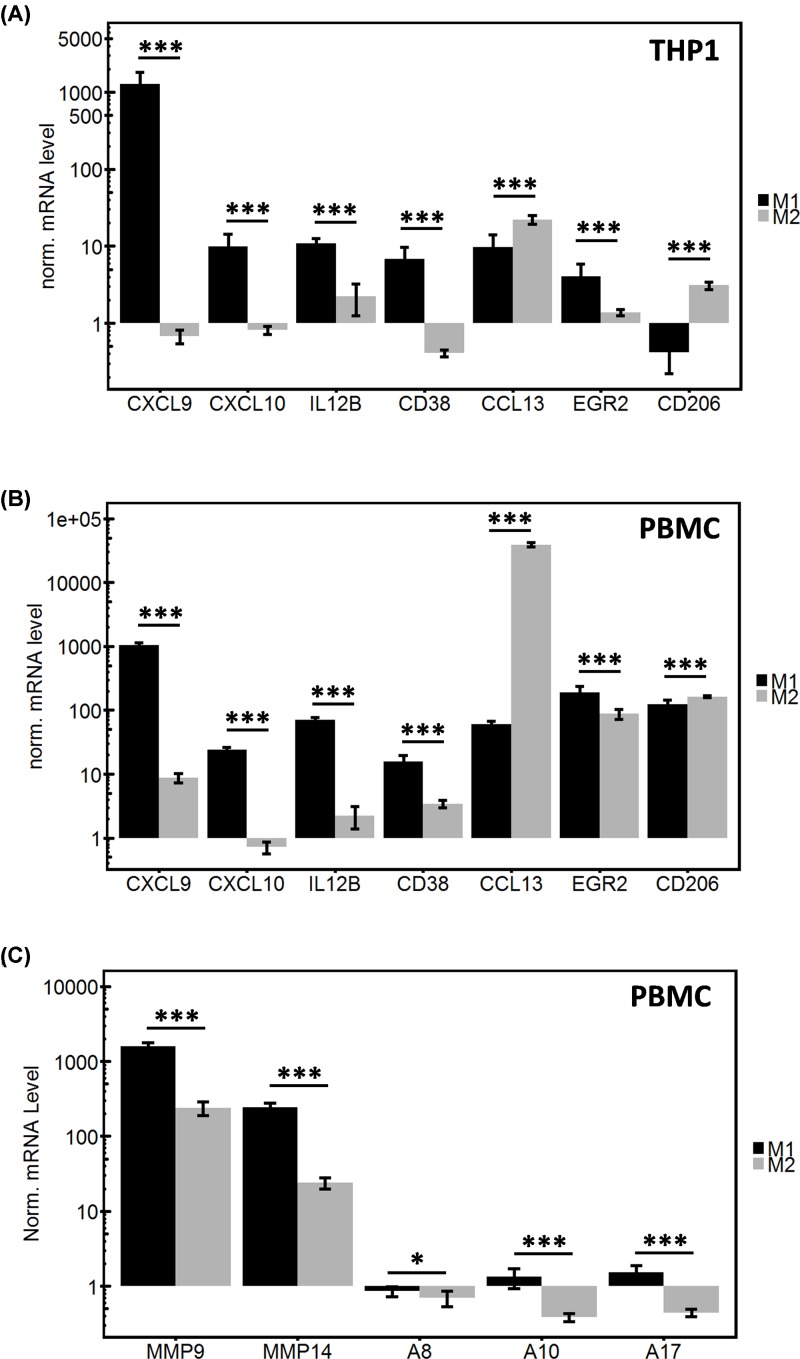
Quantitative PCR analysis of macrophage polarization markers THP1 cells were similarly polarized in either M1-like or M2-like phenotype by adding specific factors (IFNγ for M1 and IL-4 for M2). In both cases polarization was analyzed by qPCR for markers associated with a specific phenotype. qPCR was performed in triplicates and results were normalized to the reference gene (XS13) and additionally to the parental M0 cells, results are shown on a logarithmic scale. All comparisons between M1-like and M2-like cells for each specific marker were highly significant for THP1 cells (**A**) CXCL9: *P* = 0.0001375; CXCL10: *P* = 0.000265; IL12B: *P* = 0.0007499; CD38: *P* = 0.0001981; CCL13: *P* = 5.571e−06; EGR2: *P* = 0.002148; CD206: *P* = 1.064e−10) as well as for PBMCs (**B**) CXCL9: *P* = 3.257e−09; CXCL10: *P* = 1.517e−09; IL12B: *P* = 1.207e−09; CD38: *P* = 1.094e−05; CCL13: *P* = 2.588e−10; EGR2: *P* = 0.0001212; CD206: *P* = 0.0001489). The differences in protease expression between M1- and M2-like PBMC were significant in all cases (**C**) MMP9: *P* = 2.144e−08; MMP14: *P* = 2.962e−08; ADAM8: *P* = 0.04464; ADAM10: *P* = 8.289e−05; ADAM17: *P* = 2.509e−05).

THP-1 cells as monocytic cells derived from acute monocytic leukemia were polarized *in vitro* and expression level of polarized cells was compared to M0 phenotype (Figure 1A). All four analyzed M1 macrophage markers (CXCL9, CXCL10, IL12B, and CD38) showed a good discrimination depending on the polarization state, although for IL12B the discrepancy was not as high as for CXCL9, CXCL10, and CD38. In contrast, the tested M2 macrophage markers (CCL13, EGR2, and CD206) were more heterogeneous in expression. CCL13 and CD206 expression best reflected the M2-like macrophage polarization state. In addition, M1/M2 markers were tested in polarized PBMCs.

In PBMC derived macrophages all four M1 markers show high expression levels only in M1 polarized macrophages; in particular, CXCL10 showed the highest difference when comparing M1 with M2 polarized macrophages. For M2 markers tested, the results were more heterogeneous. Whereas EGR2 and CD206 markers showed no difference comparing M1 with M2 polarization, only CCL13 was identified as the most distinct M2 marker (80 for M1 vs. 30000 for M2, Figure 1B).

From these experiments we conclude that CXCL10 and CCL13 are the most informative markers to discriminate between M1- and M2-like macrophage populations, respectively, given their expression levels in THP1 and PBMC derived macrophages. Therefore, these two markers were used in the following experiments with the aim to describe the global microglia/macrophage polarization state in GBM.

Given these validated macrophage polarization markers, we also analyzed the differences in protease expression levels in correlation to M1/M2 polarization in PBMCs. Protease genes were selected by their reported involvement in GBM pathology and their association with patient survival as analyzed in the global gene expression database TCGA (www.tcgabrowser.ethz.ch, median survival data). TCGA data analysis of 159 GBM patients revealed either an association with no significant effects on overall survival for ADAM10 (384 d for high vs. 432 d for low expression, *P* = 0.6036) and for ADAM17 (442 d for high vs. 405 d for low, *P* = 0.5412). Moreover, proteinases ADAM8 (360 d for high vs. 454 d for low, *P* = 0.0685) and MMP14 (375 d high vs. 460 d low, *P* = 0.0824) are significantly associated with an impaired patient prognosis when highly expressed. A similar tendency, although not statistically significant, was observed for MMP9 (360 d for high vs. 468 d for low, *P* = 0.2371). Thus, we hypothesized that proteinase gene expression might be clustered with a distinct microglia/macrophage phenotype. M1-like macrophages compared to M2-like macrophages show higher expression levels for all protease genes analyzed: *MMP9, MMP14, ADAM8, ADAM10*, and *ADAM17*. The expression levels of MMP genes were generally higher than expression of ADAM protease genes (Figure 1C).

### An M2-like microglia/macrophage subtype is predominantly expressed in GBM

**Figure 2:**
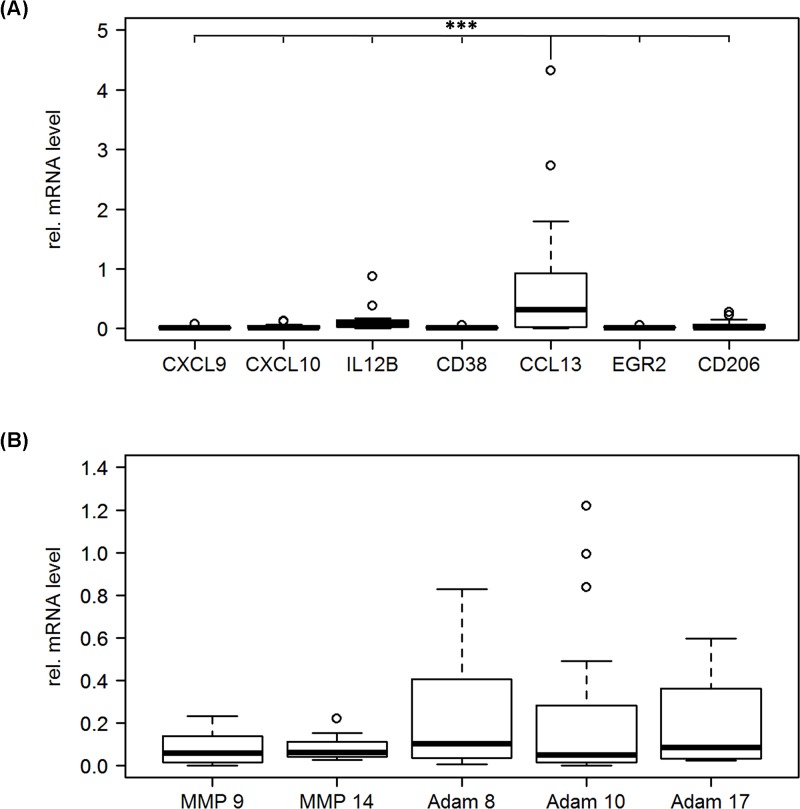
Expression of macrophage markers and protease genes in GBM tissue GBM tissue samples were analyzed by qPCR for the expression of macrophage markers (**A**) and proteases (**B**). qPCR was performed in triplicates and results were normalized to the reference gene (XS13). Note that CCL13, the most promising M2 macrophage marker clearly shows the highest expression level, this difference in expression was significant comparing CCL13 to all other markers (CXCL9/CCL13: *P* = 0.0000115; CXCL10/CCL13: *P* = 0.0000175; IL12B/CCL13: *P* = 0.0004864; CD38/CCL13: *P* = 0.0000119; EGR2/CCL13: *P* = 0.0000123; CD206/CCL13: *P* = 0.0000434), all other comparisons were not significant. Differences in protease gene expression levels were also not significant in all cases.

The macrophage markers described above were used to analyze macrophage subtypes in GBM tissue in a patient cohort of 20 patients (Figure 2A). The M2 macrophage marker CCL13 showed significantly higher expression levels than all M1 macrophage markers. This supports the notion that the M2-like macrophage phenotype is the predominant one in GBM.

To further investigate a possible link between macrophage phenotype and protease expression, qPCR analysis for expression levels of MMP9, MMP14, ADAM8, ADAM10, and ADAM17 were performed. The results show relevant mRNA expression levels for all proteases investigated (Figure 2B).

### MMP and ADAM protease genes are expressed in particular clusters

**Figure 3:**
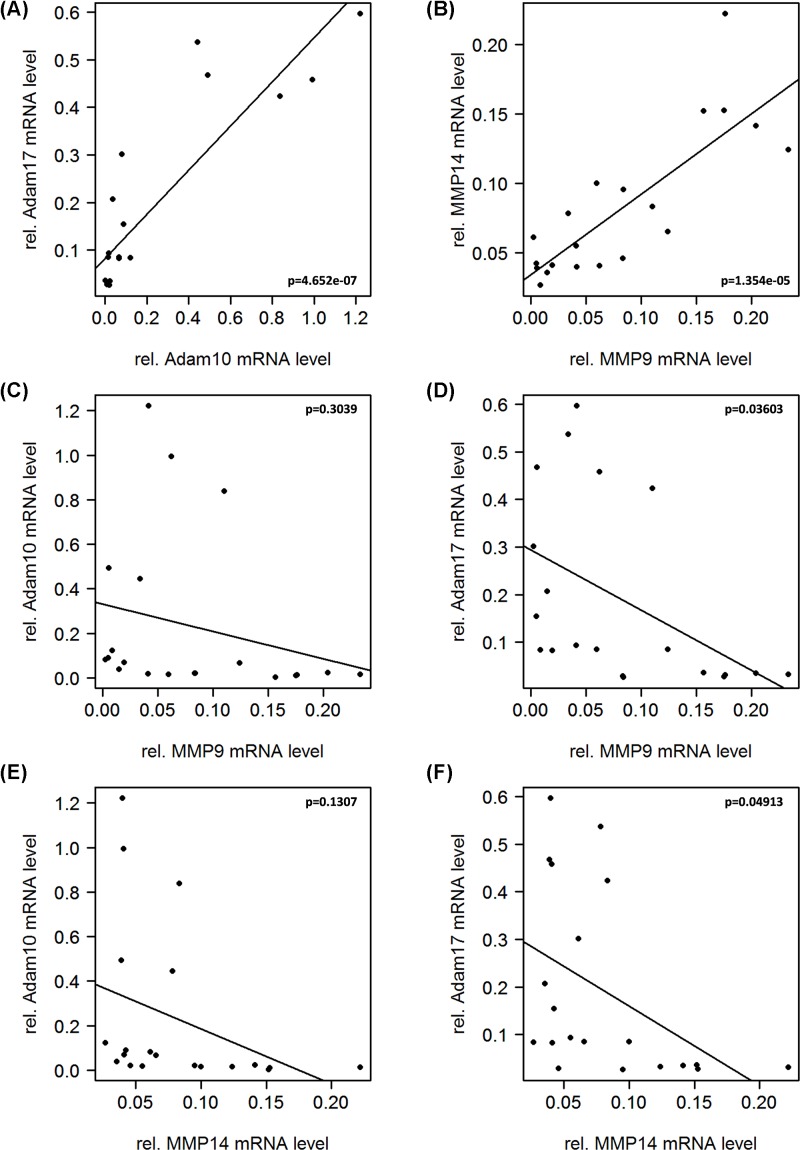
Correlation analysis of protease genes in GBM tissue Correlation of MMPs 9 and 14, ADAM8, 10, and 17 amongst themselves reveals similar expression profiles for (**A**) MMP9 and MMP14 (*P* = 1.354e−05) as well as (**B**) ADAM10 and ADAM17 (*P* = 4.652e−07). Whereas the Pearson correlation across the assayed members of the MMP and ADAM families reveals that they are regulated in an inverse manner. This trend is clearly perceptible for all correlations (**C**–**F**), however it is only significant for the association between MMP14 and ADAM17 (**F**). (MMP9/ADAM10: *P* = 0.3039; MMP9/ADAM17: *P* = 0.03603; MMP14/ADAM10: *P* = 0.1307; MMP14/ADAM17: *P* = 0.04913).

Clustering of the protease genes *MMP9, MMP14, ADAM8, ADAM10*, and *ADAM17* was compared based on qPCR results. We found that from both MP families, two members, ADAM10/ADAM17 and MMP9/MMP14, showed similar expression profiles. As seen from the Pearson correlations (Figure 3A,B), this association is significant in both cases with *P*<0.001. Whereas this correlation is proportional, the two families seem to be inversely correlated, leaving a high MMP expression with a low ADAM expression and vice versa (Figure 3C–F).

**Figure 4:**
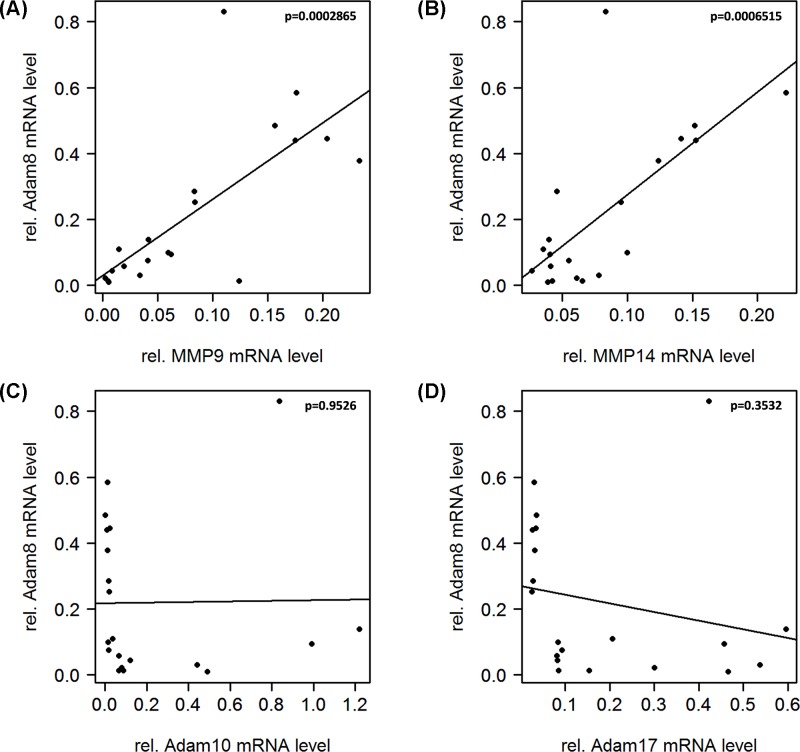
Correlation analysis of *ADAM8* with *MMP* and *ADAM* genes Different from ADAM10 and ADAM17, ADAM8 showed a positive correlation with the investigated MMPs, which was highly significant in both cases (MMP9/ADAM8: *P* = 0.0002865; MMP14/ADAM8: *P* = 0.0006515). ADAM8 seems to be clustering with MMP9 and MMP14 rather than ADAM10 and ADAM17 (ADAM10/ADAM8: *P* = 0.9526; ADAM17/ADAM8: *P* = 0.3532).

Interestingly, the described correlation was not observed for ADAM8, which exerted an expression profile distinctly different from those observed for ADAM10 and ADAM17. A completely opposite trend was observed in the correlation of ADAM8 with MMPs, concluding that a high expression in MMP9 and MMP14 is significantly correlated (*P*<0.001) with a high expression of ADAM8 in GBM (Figure 4A,B). Moreover, no clear association was found between ADAM8 expression and the expression levels of ADAM10 or ADAM17 (Figure 4C,D).

### ADAM10 and ADAM17 expression linked to M1-like, MMP9 and MMP14 expression linked to M2-like microglia/macrophage

**Figure 5:**
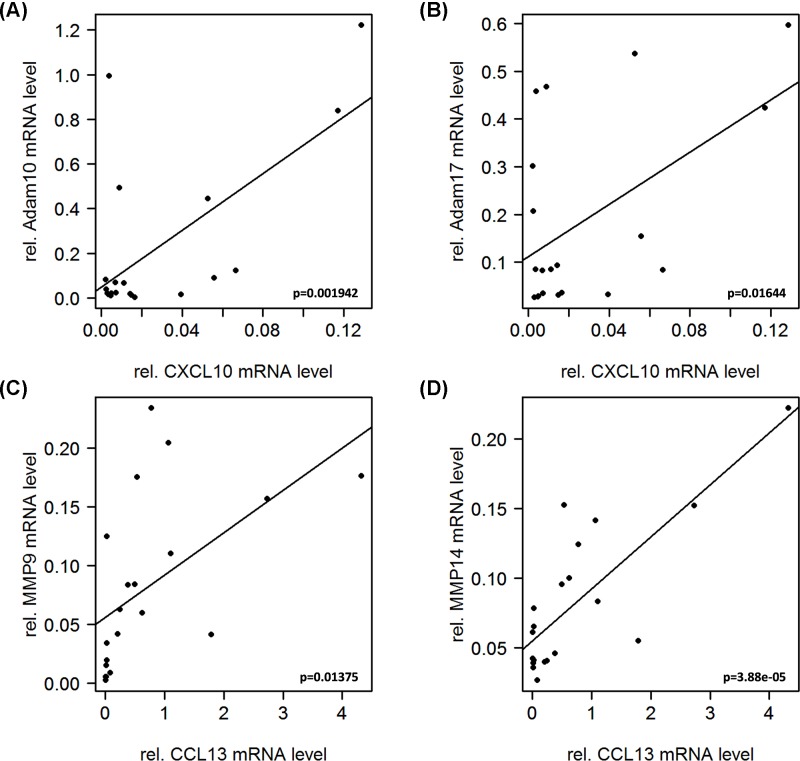
Correlation analysis of M1/M2-like markers with protease gene expression The correlations shown support the notion that ADAM10 and 17 are expressed by M1-like macrophages, while MMP9 and MMP14 are mainly expressed in M2-like macrophages, as they show significant correlation with the respective markers (CXCL10/ADAM10: *P* = 0.001942; CXCL10/ADAM17: *P* = 0.01644; CCL13/MMP9: *P* = 0.01375; CCL13/MMP14: *P* = 3.88e−05).

Since the goal of this study was to provide clues on expression levels of proteases to a specific microglia/macrophage polarization type, macrophage marker expression was correlated to protease expression. Our findings indicate that ADAM10 and ADAM17 are mainly associated with M1-like microglia/macrophage, as both proteases correlated significantly (for ADAM10 *P*<0.01 and for ADAM17 *P*<0.05) with the M1 macrophage marker CXCL10 (Figure 5A,B). In contrast, MMP9 and MMP14 seem to be expressed by M2-like microglia/macrophage demonstrated by a significant (for MMP9 *P*<0.05 and for MMP14 *P*<0.001) linkage to CCL13 expression (Figure 5C,D). Confirming our results, an opposite trend was observed for the correlation between ADAM10/CCL13 and ADAM17/CCL13 as well as MMP9/CXCL10 and MMP14/CXCL10 (Supplementary Figure S1). Interestingly the observed linkage of the ADAMs 10 and 17 to M1 macrophages was not observed for ADAM8, which showed a significant correlation to both M1 (CXCL9, IL12B) and M2 (CCL13) macrophage markers (data not shown).

### ADAM10 and ADAM17 expression levels are linked to improved prognosis, whereas MMP9 and MMP14 expression levels are associated with shorter overall survival

We then asked if the expression of proteases have a clinical relevance by affecting the outcome of patients with GBM. To this end, we correlated the expression of ADAMs 10 and 17 as well as MMPs 9 and 14 with the survival of patients.

**Figure 6:**
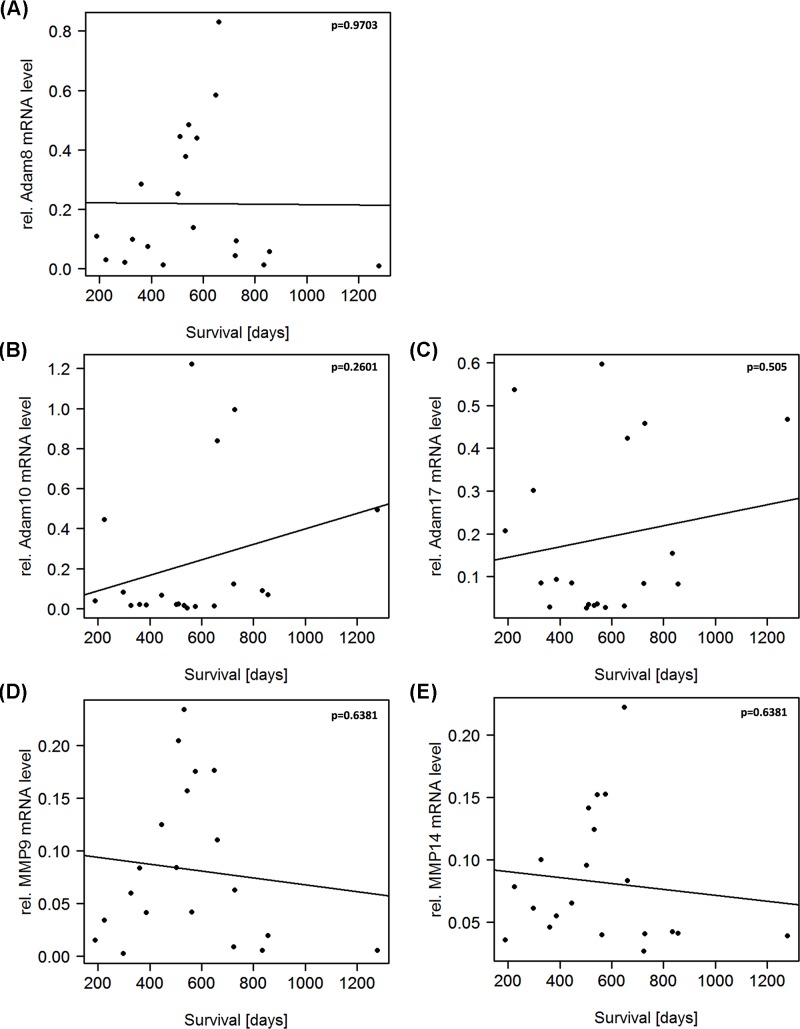
Correlation analysis of protease gene expression with survival of GBM patients Although not statistically significant trends were observed that protease expression impacts on the overall survival of GBM patients (ADAM8/Survival: *P* = 0.9703; ADAM10/Survival: *P* = 0.2601; ADAM17/Survival: *P* = 0.505; MMP9/Survival: *P* = 0.6381; MMP14/Survival: *P* = 0.6318). ADAM10 and ADAM17 are linked to a better prognosis, fitting the result that they are expressed by anti-tumorigenic M1 macrophages. In contrast, high expression of MMP9 and MMP14 is associated with a shorter overall, also fitting the previous result that they are expressed by pro-tumorigenic M2 macrophages.

In line with our findings that ADAM10 and ADAM17 are expressed by M1 macrophages, we see a trend, although not statistically significant, that a high ADAM10 and ADAM17 expression is linked to a longer survival (Figure 6B,C). This is what was to be expected since M1 macrophages have been reported to be anti-tumorigenic. In contrast to this, M2 macrophages, which we found to be associated with the expression of MMP9 and MMP14, are thought to have a pro-tumorigenic effect. It is therefore fitting that MMP9 and MMP14 expressions are linked to shorter overall survival (Figure 6D,E). Furthermore, for ADAM8 no trend of an effect on survival was observed (Figure 6A), and likewise, no clear linkage to one particular macrophage phenotype was seen. In accordance with their proposed molecular signature, we were able to correlate patient survival data with the expression levels of the respective macrophage markers (see Supplementary Figures S2 and S3) in our cohort.

## Discussion

Here we demonstrate that the tumor microenvironment in GBM patient cohort can be profiled by M1-like and M2-like microglia/ macrophage markers CXCL10 (M1-like) and CCL13 (M2-like) and by a subset of metalloprotease genes. Our findings on GBM tumors are supported by the correlation of M1/M2 markers with patient survival and support the notion that, although not statistically significant, a predominant M2-like microglia/macrophage polarization is associated with an impaired prognosis whereas a predominant M1 polarization is associated with better overall prognosis of GBM patients. These results were obtained from a patient cohort of 20 well-documented GBM patients so that a larger patient cohort could lead to a higher significance of our results. In previous studies, a microglia/macrophage polarization type distinct from M1/M2 was reported to be associated with GBM. However, these microglia/macrophage polarization data are derived from GL261 injected C57BL/6 mice [18] and might not be transferable to human GBM.

The distinct expression patterns of genes associated with M1/M2 polarization and patient survival provides a novel view on the possible function of the proteases analyzed in the progression of GBM. In earlier studies, ADAM10 functions in GBM were associated with enhanced GBM cell migration by N-Cadherin cleavage [10], whereas ADAM17 was described in GBM stem cell differentiation, migration and invasion [1]. The combined presence of ADAM10 and ADAM17 was described to be important for immunogenicity of GBM stem cells [21] and differentiation of GBM sphere-forming cells [17]. Despite these important functions in GBM stem cells, these two ADAM proteases do not seem to be clinically relevant, as their expression levels are not (ADAM10) or even positively (ADAM17) correlated with GBM patient survival, as extracted from TCGA datasets. This finding is in accordance with the association of ADAM10 and ADAM17 to a merely M1-like phenotype. In contrast, TCGA data as well as data defining markers associated specifically with microglia/macrophages in GBM [18] demonstrate that MMP9 [2,3] and MMP14 [13] could be target proteases in GBM. It was described earlier that MMP9 is instrumental for active suppression of apoptosis in GBM cells [2] and for induction of GBM cell migration by cleavage of CD44 [3]. Also, MMP14 was identified as a protease important in microglia so that a therapy approach was conducted to explore the efficacy of minocycline in GBM mouse models [13]. We found that MMP9 and MMP14 are negatively correlated with GBM patient survival and associated with the markers to define a more M2-like microglia/macrophage phenotype.

Despite limitations of this study with regard to patient number and solely gene expression analyses, we provide evidence for the validity of microglia/macrophage markers CXCL10 and CCL13 in GBM tissue with the GBM patient cohort presented here. This, in conjunction with the observed protease expression profiles, provides a suitable system to determine patient prognosis and potentially define those patients that could benefit from a therapy that has recently attracted attention in order to repolarize GAMs. This appears feasible by employing two different approaches: i) a bispecific antibody directed against angiopoetin-2 (Ang-2) and vascular endothelial growth factor (VEGF) [10], or ii) a combination of different compounds, collectively termed as ‘TriCurin’, a liposomal combination of Curcumin, Epigallocatechin gallate, and Resveratrol [14]. Mechanistically, curcumin as the major compound of ‘TriCurin’ seems to activate natural killer cells for subsequent repolarization of tumor-associated microglia/macrophages [15]. It is interesting to note that both approaches are effective to repolarize tumor-associated microglia/ macrophages by either interfering with tumor angiogenesis (bispecific antibody) or by eliminating GBM cells and GBM stem cells.

## Conclusion

Our study establishes M1-/M2-like markers CXCL10 and CCL13 for informative and reliable detection of GBM associated microglia/macrophage polarization in conjunction with a defined protease profile as molecular determinants for GBM progression. These findings can be converted into a diagnostic mean to predict patient prognosis, therapy response, and could aid to define those patients for which reprogramming of GAMs can be beneficial.

## Supporting information

**Supplementary Figure S1:** 

**Supplementary Figure S2:** 

**Supplementary Figure S3:** 

## Abbreviations

ADAM: a disintegrin and metalloprotease
GAM: Glioma-associated microglia/macrophage
GBM: glioblastoma
IL: interleukin
MMP: matrix metalloproteinase
PBMC: peripheral blood monocytic cell
qPCR: quantitative PCR
