# Supplementary figures and images for: Molecular profiling of the tumor microenvironment in glioblastoma patients: correlation of microglia/macrophage polarization state with metalloprotease expression profiles and survival
Source: Biosci Rep. 2019 Jun 20;39(6):BSR20182361. doi: 10.1042/BSR20182361 (PMC6616040; doi:10.1042/BSR20182361)

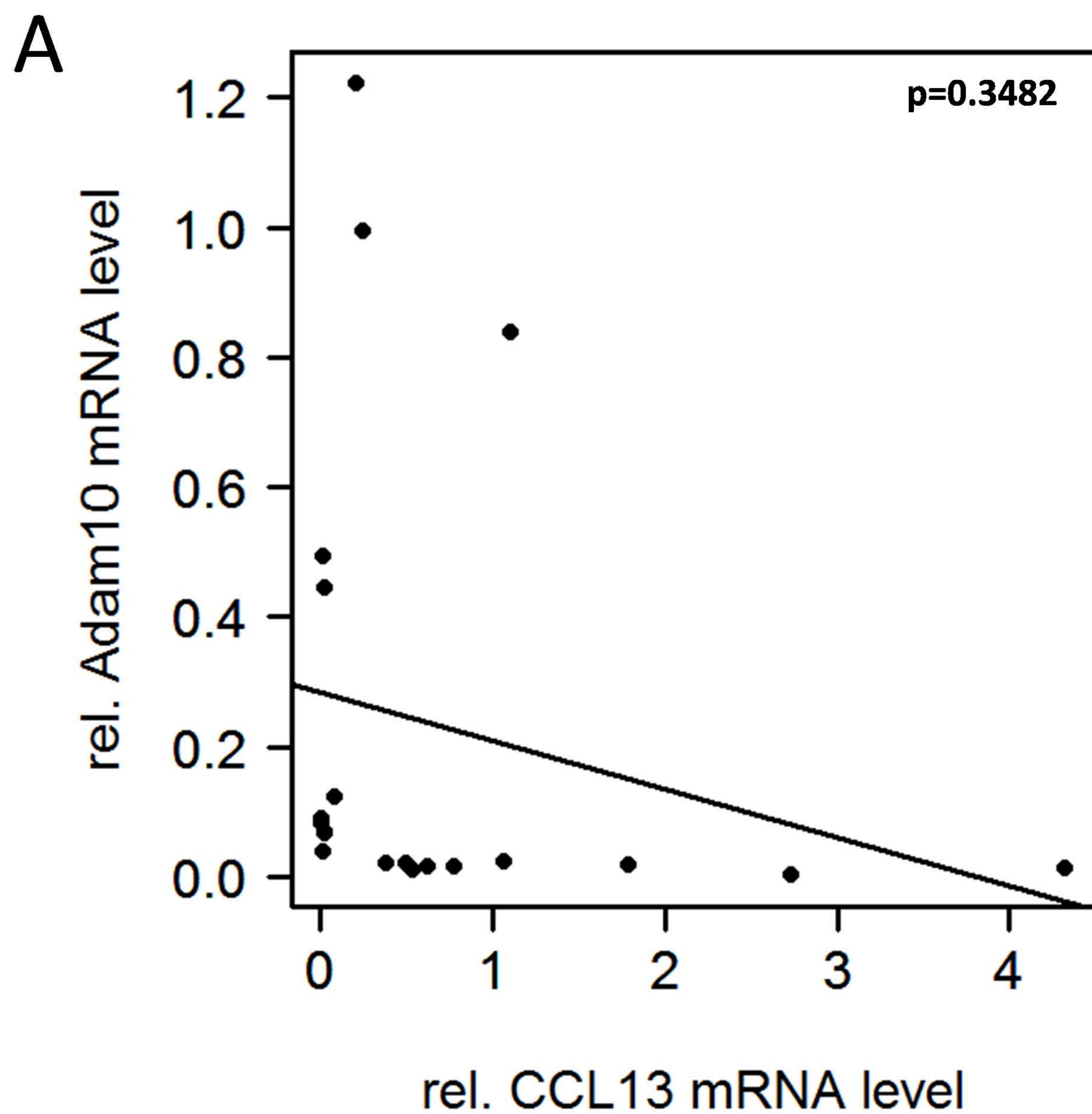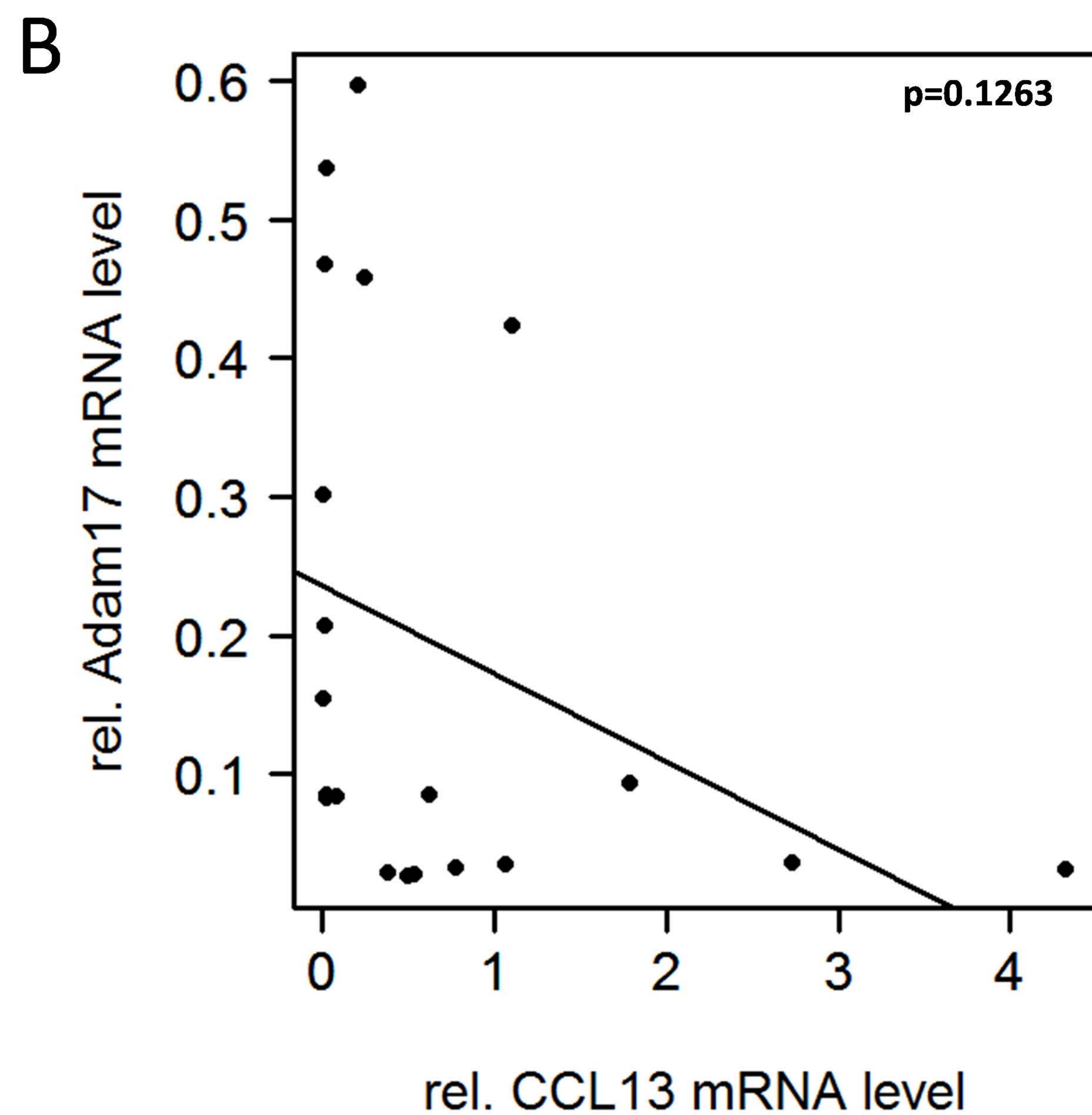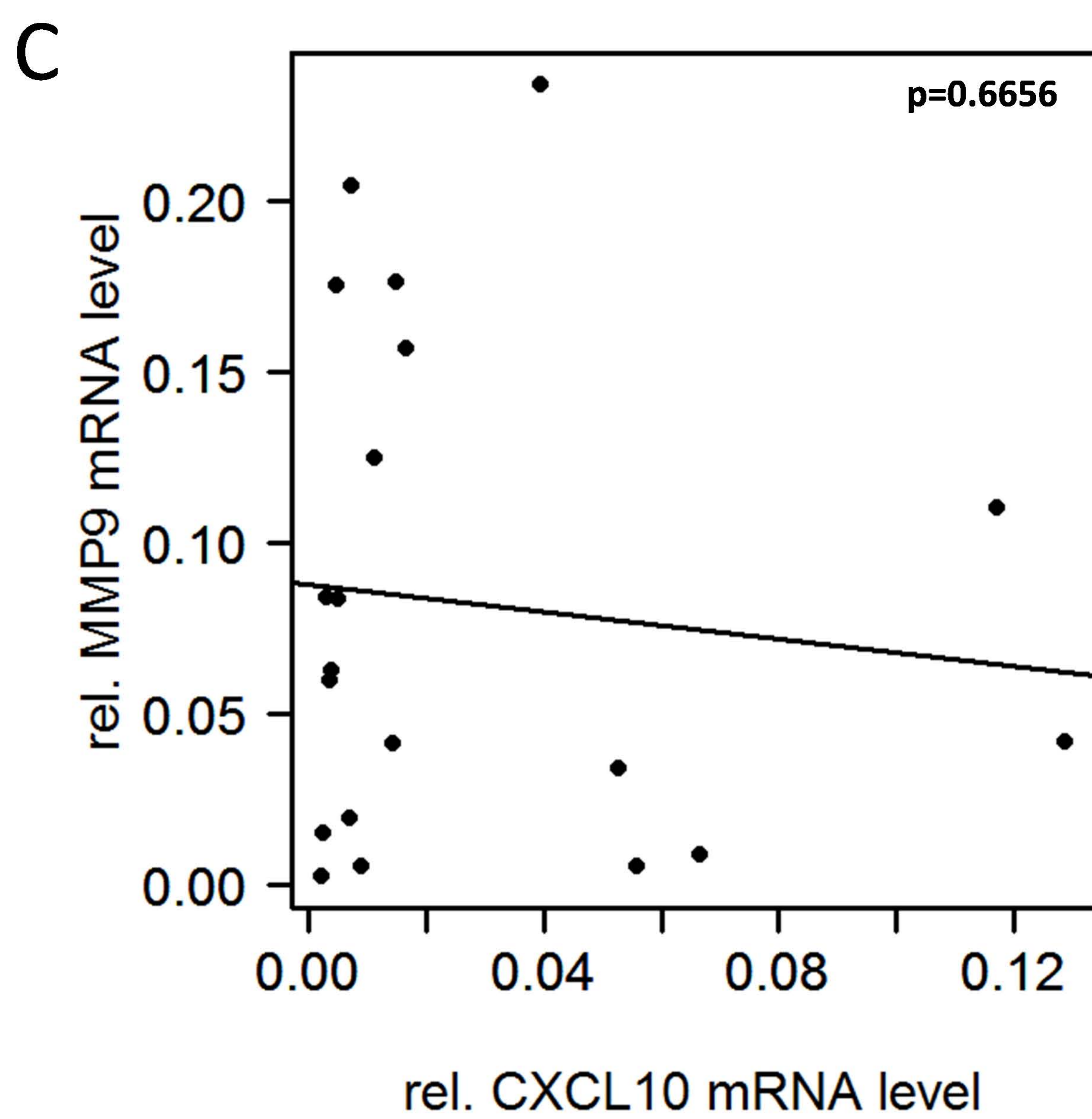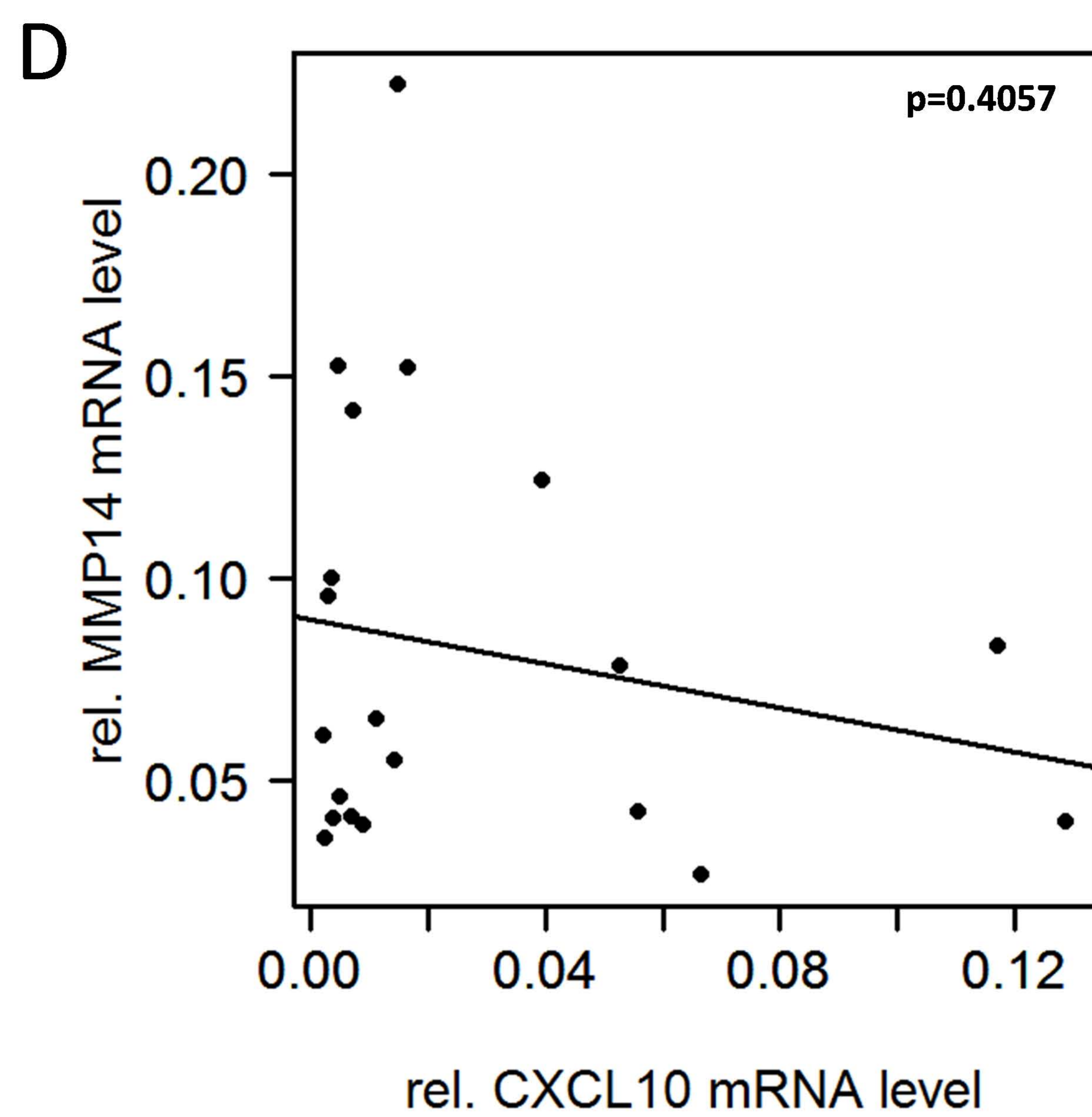

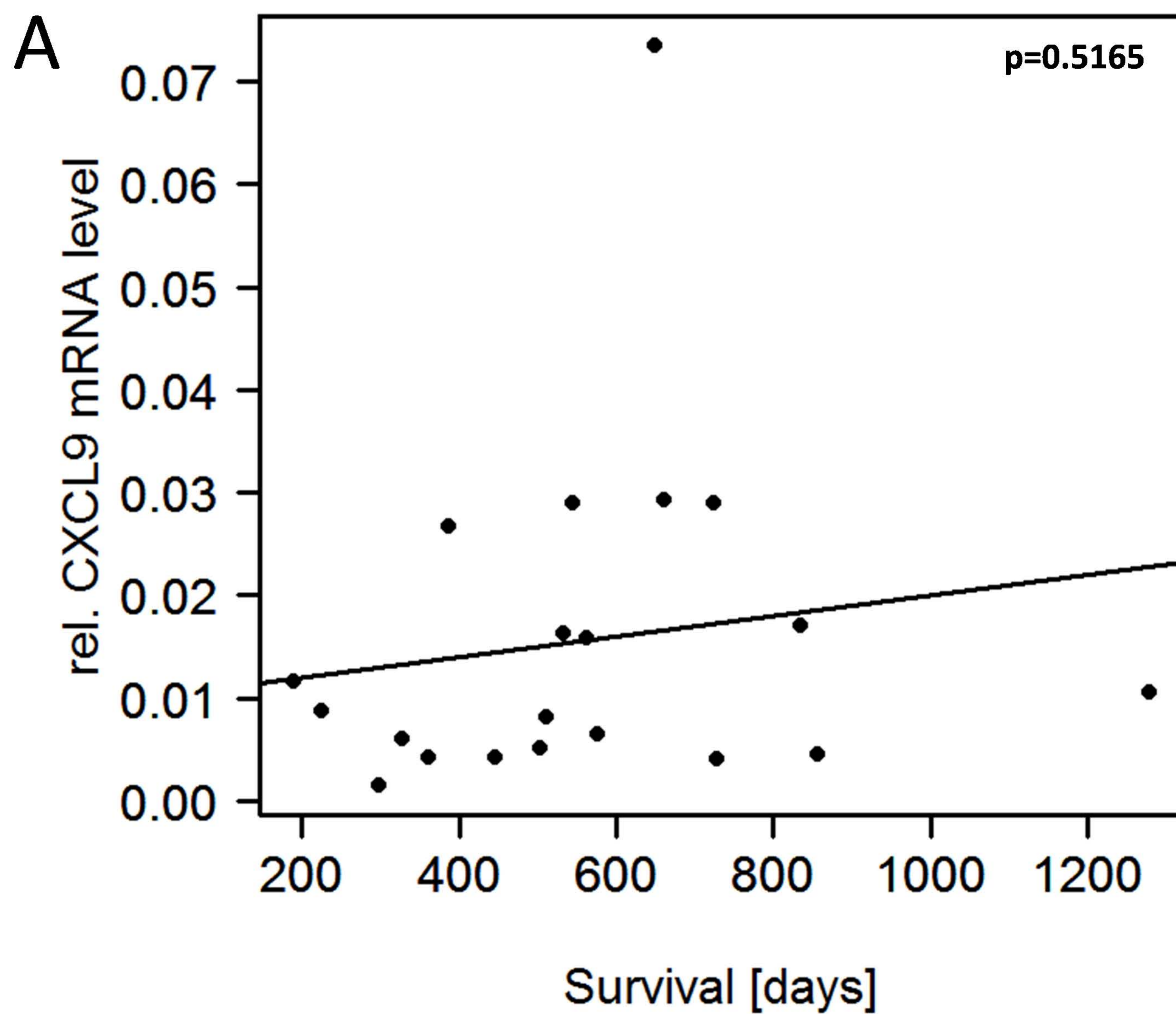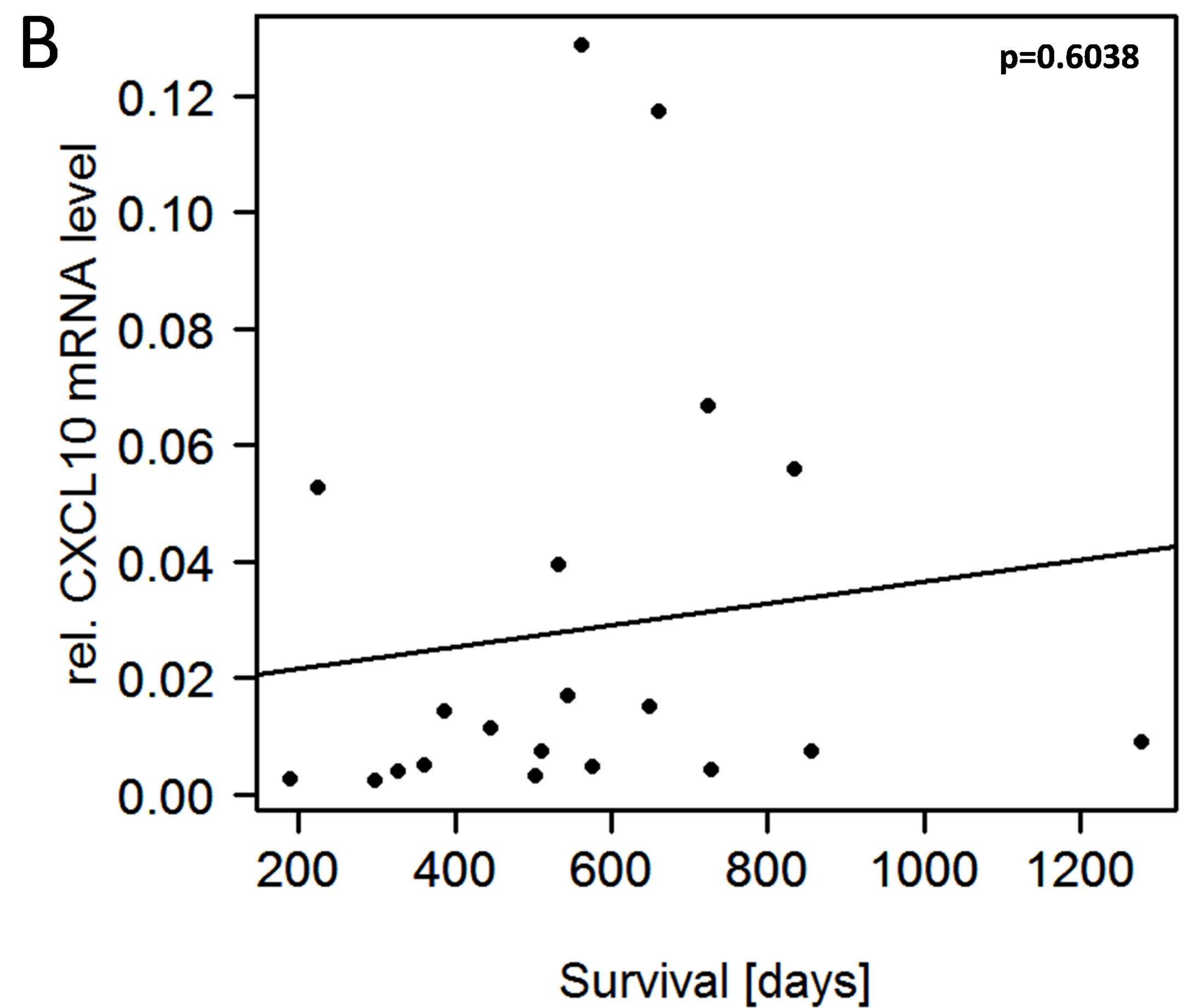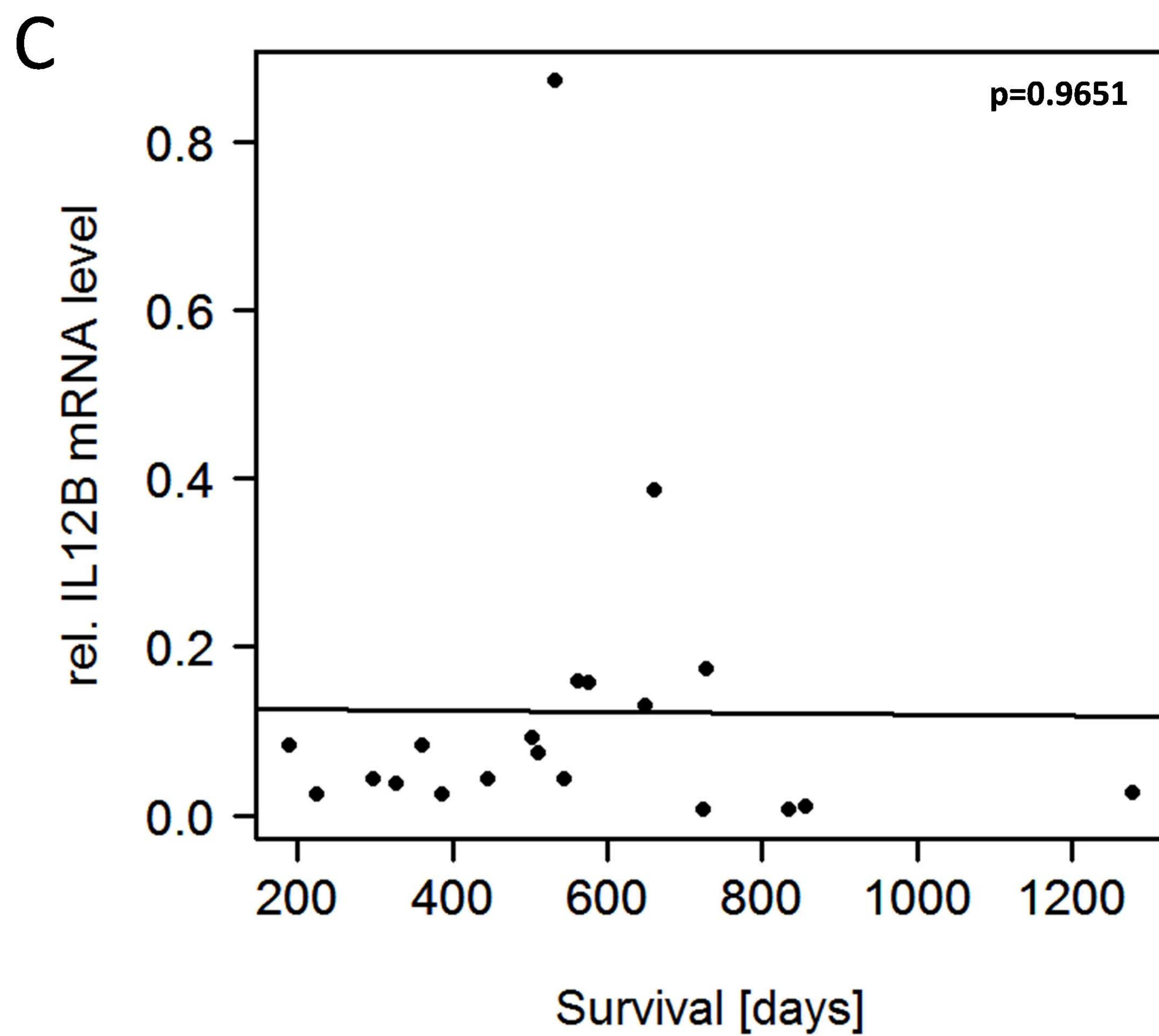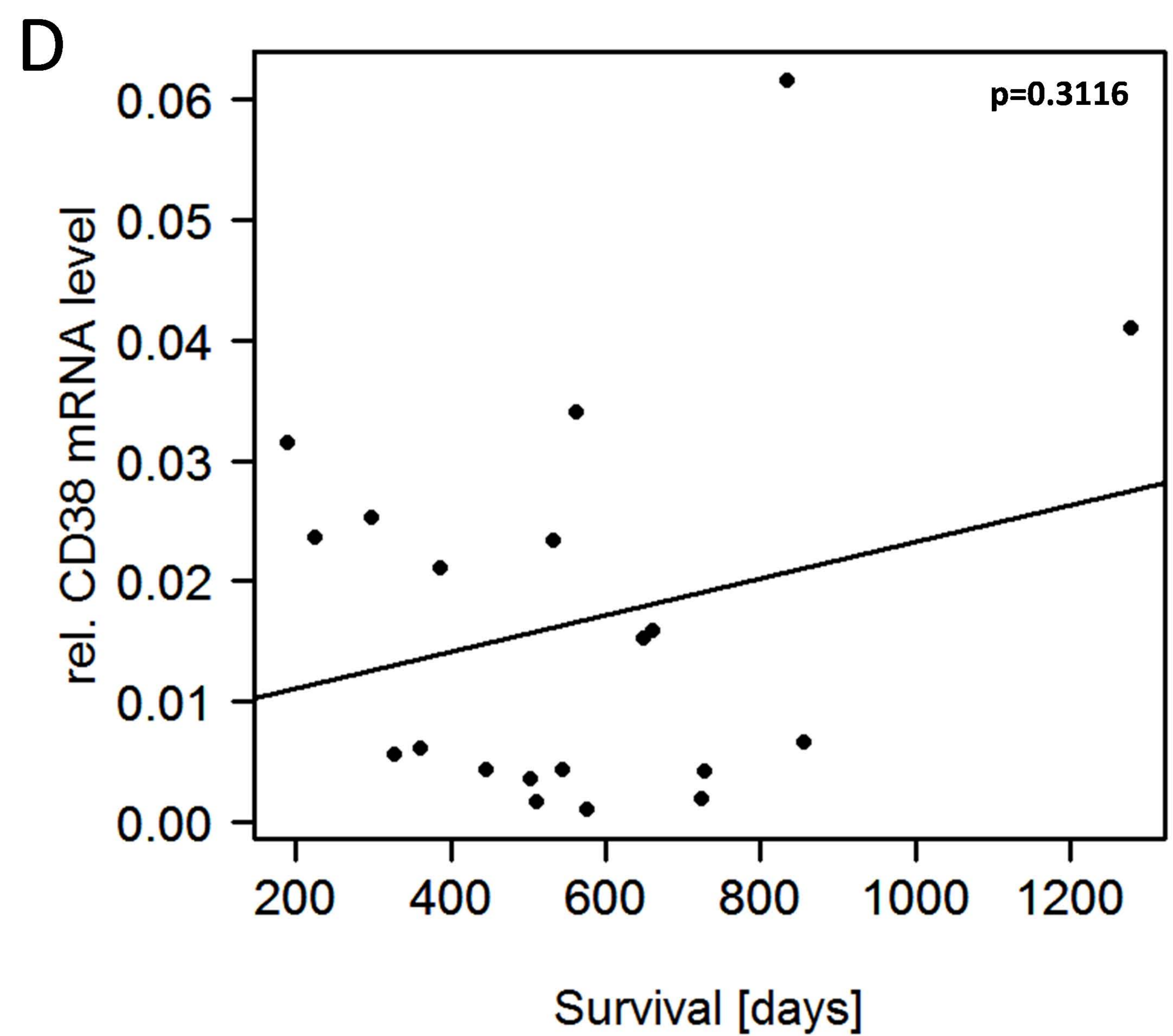

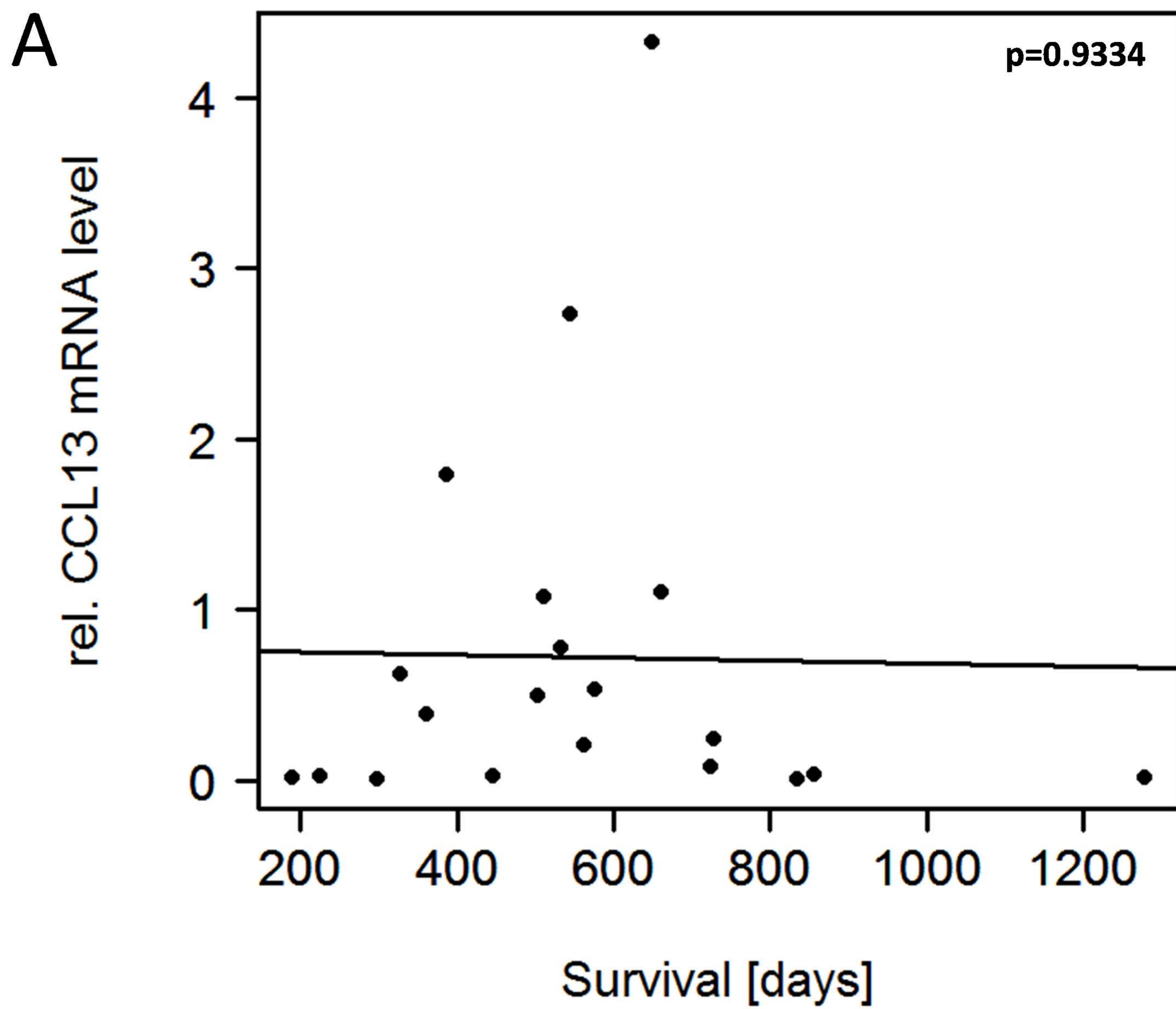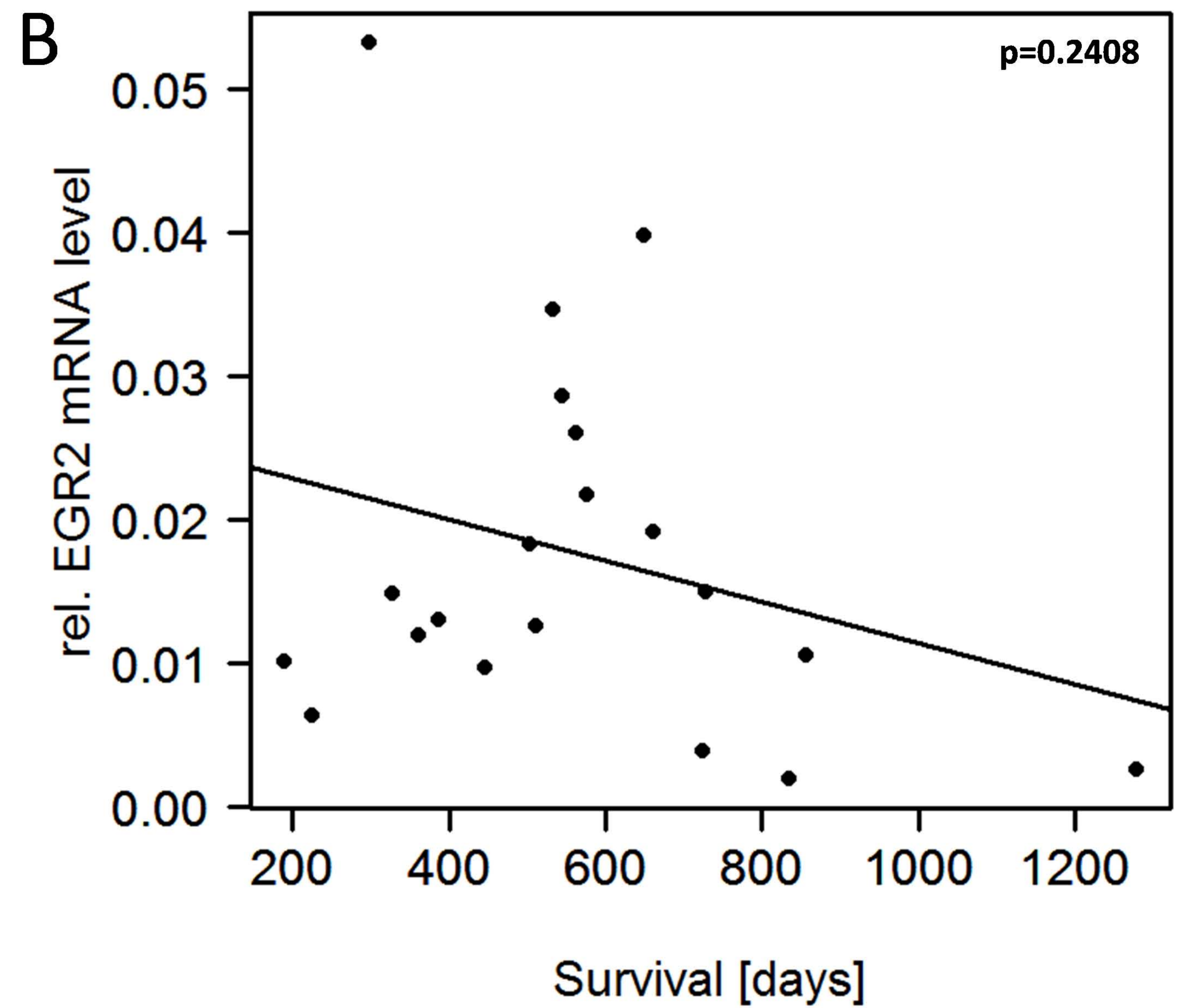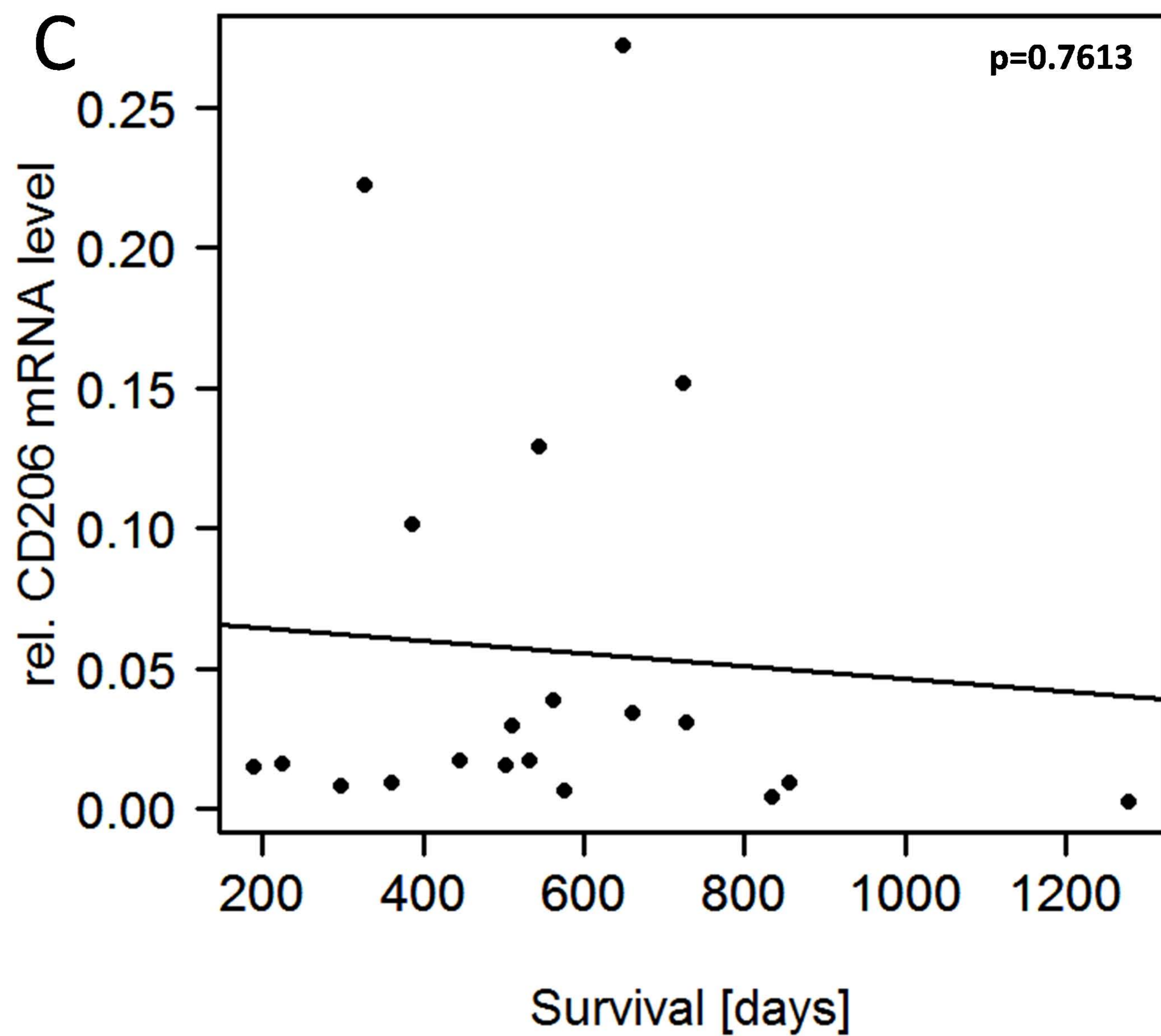

Supplement: Supplementary file 1 [file bsr20182361_Supp1.pdf]
